# Supplementary material for: Arabinose Plays an Important Role in Regulating the Growth and Sporulation of Bacillus subtilis NCD-2
Source: Int J Mol Sci. 2023 Dec 14;24(24):17472. doi: 10.3390/ijms242417472 (PMC10744016; doi:10.3390/ijms242417472)
Supplement: Supplementary file 1 [file ijms-24-17472-s001.zip › ijms-2701039-supplementary.pdf]

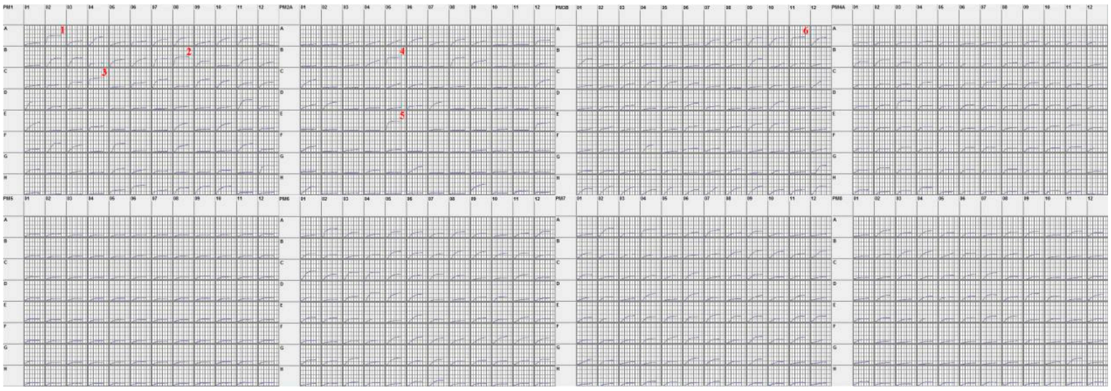

Figure S1. Growth kinetics of *B. subtilis* NCD-2 in the individual wells of PM1-8. The name of a plate is labeled in the upper left corner of each individual figure. The numbers 1-6 (red) indicate L-arabinose, D-xylose, D-ribose, D-arabinose, D-glucosamine and cysteine, respectively. A–H indicate the number of rows in a plate; 1–12 (black) indicate the number of columns in a plate. Each well displays time on the X-axis and redox signal intensity on the Y-axis.

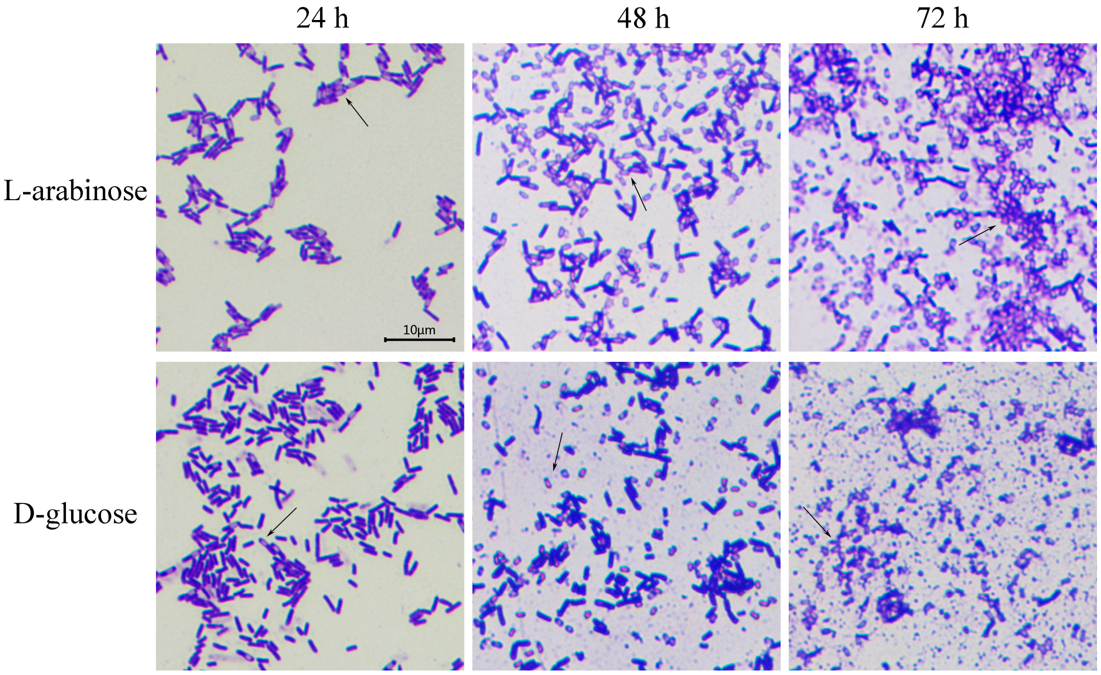

Figure S2. Proportion of spores of strain NCD-2 in M9 medium with L-arabinose/D-glucose as the carbon sources, at 24 h, 48 h and 72 h post-inoculation. The black arrows indicate spores formed by strain NCD-2 .

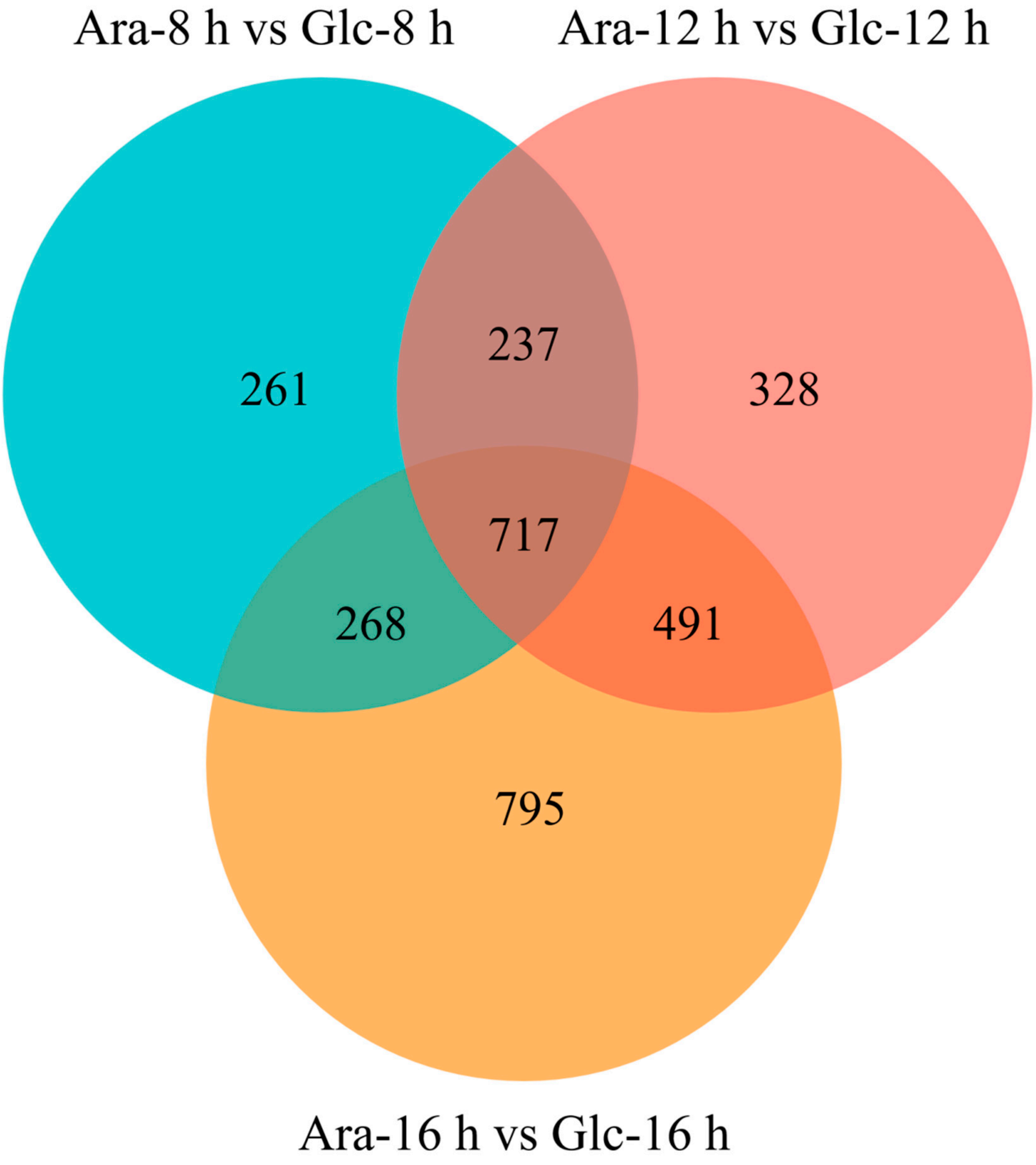

Figure S3. Venn diagrams depicting the numbers of shared differentially expressed genes (DEGs) in strain NCD-2 at 8 h, 12 h and 16 h of culture.

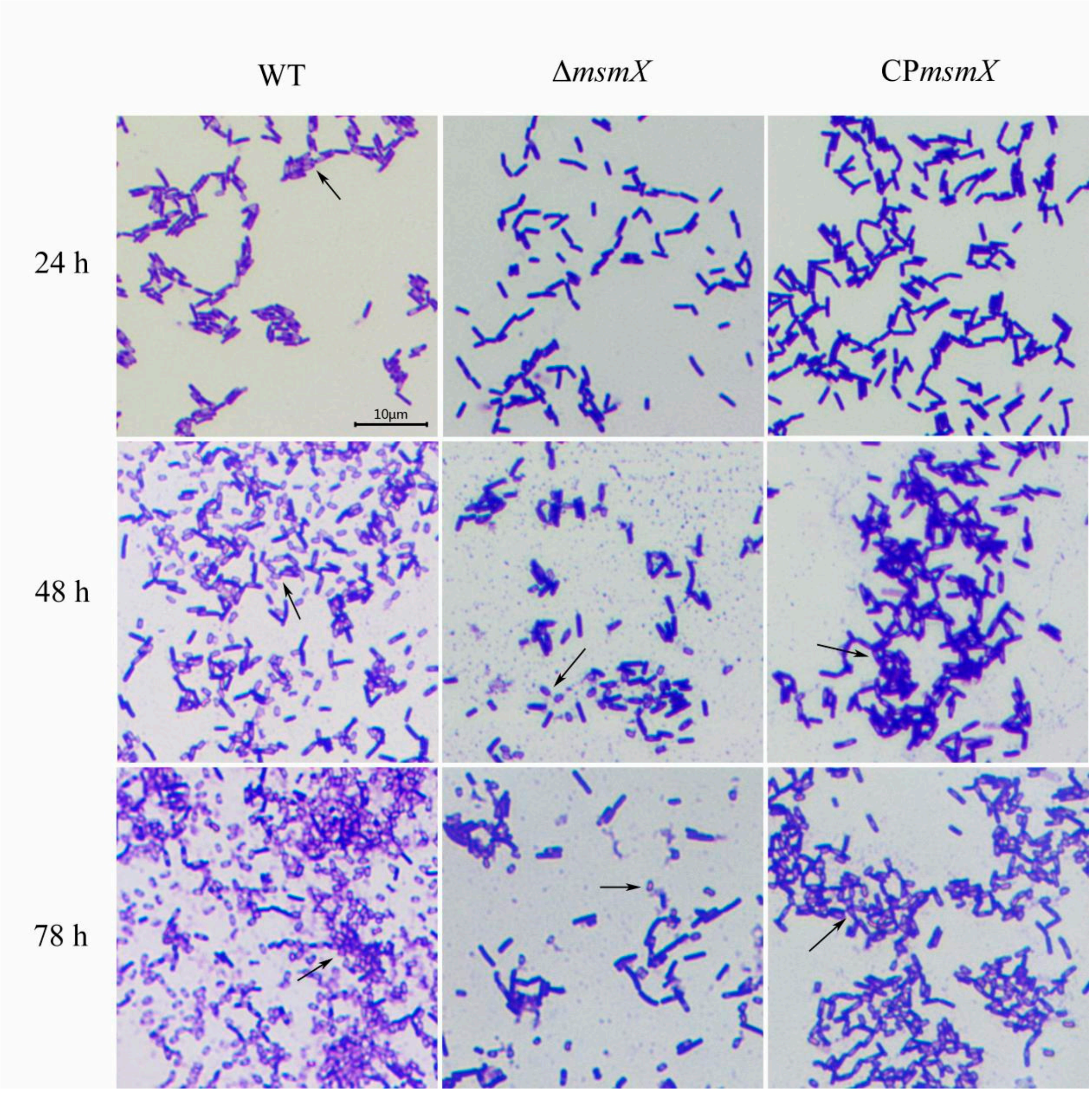

Figure S4. Proportion of spores of strain NCD-2 wild type (WT), *msmX*-null mutant (Δ*msmX*) and its complemented strain (CP*msmX*) in M9 medium with L-arabinose as the carbon source, at 24 h, 48 h and 72 h post-inoculation.

Table S1. Verification of differently express genes by RT-qPCR

Gene	log_2(Ara/Glc) (transcriptome)			log_2(Ara/Glc) (RT-qPCR)		
	8 h	12 h	16 h	8 h	12 h	16 h
cotY	5.16	7.90	9.47	5.76	8.83	9.01
dpaA	6.30	6.84	4.81	5.50	6.42	5.10
dpaB	5.76	6.12	4.21	5.12	5.98	4.37
msmX	2.96	1.35	-[a]	3.14	1.51	-
sspA	7.23	3.14	1.67	8.11	3.51	2.01
sspB	7.40	3.43	2.66	7.71	3.25	3.05
tuaC	1.65	1.12	5.15	1.78	1.35	4.82
spoIIQ	5.69	1.78	-1.82	5.47	1.33	-2.04
spo0E	-	-1.75	-1.93	-	-2.21	-2.34
sdpC	-1.23	-5.57	-6.81	-1.77	-6.08	-7.71
leuA	-1.34	-1.03	-2.98	-1.19	-1.28	-3.22
ilvC	-1.45	-1.36	-3.80	-1.73	-1.39	-3.77
srfAA	-2.46	-1.88	-2.41	-2.89	-1.79	-2.50
ppsC	-3.00	-4.75	-4.66	-3.25	-4.17	-4.23

a: "-" means no significant difference in gene expression between L-arabinose (Ara) treatment and D-glucos (Glc) treatment

Table S2. Primers used in this study

Primer	Sequence (5′ to 3′)	Function
cotY-F	AAGGCGGATTGTTCTCCA	qRT-PCR for *cotY*
cotY-R	TCTGGTGACAGGCACTGAAT	
dpaA-F	CAGGAGAAGGTGTCGTATCG	qRT-PCR for *dpaA*
dpaA-R	TCATCCCGCTCAAACAGT	
dpaB-F	CGATTGTAAAGGCAGAACCT	qRT-PCR for *dpaB*
dpaB-R	GAGATACCCAGAACGACAGG	
msmX-F	GACCTCTTTGCCGATGTAGC	qRT-PCR for *msmX*
msmX-R	GGACGCCGAAGGATGTTTA	
sspA-F	ACTCAGGTAACAGCAACAACC	qRT-PCR for *sspA*
sspA-R	TCCTCCAACAGAACCGTT	
sspB-F	TAACCTTGGAGCGGACACT	qRT-PCR for *sspB*
sspB-R	TGCTGCTGAGCGAAAGAT	
tuaC-F	CGTAGGTTCTGCGTCAGTTG	qRT-PCR for *tuaC*
tuaC-R	GATTTCCTCTTTCGTCTCCTCA	
spoIIQ-F	AAGAGAAAGAAGCAGCACTCG	qRT-PCR for *spoIIQ*
spoIIQ-R	CGCTCAGAGAAGCAGAGACA	
Spo0E-F	AAGAGAAAGAAGCAGCACTCG	qRT-PCR for *Spo0E*
Spo0E-R	CGCTCAGAGAAGCAGAGACA	
sdpC-F	CGATTGACAGCAAAGACCC	qRT-PCR for *sdpC*
sdpC-R	GCATAAAGCCCACAAGATGG	
leuA-F	TTCGTGATGGTGAACAGTCC	qRT-PCR for *leuA*
leuA-R	TCTTGCGATTTCCTGAACAG	
ilvC-F	CGGCGGATGTAGATGTATTCT	qRT-PCR for *ilvC*
ilvC-R	GCTTTGTCTCTTGCTTCTCCA	
srfAA-F	CGGCGGATGTAGATGTATTCT	qRT-PCR for *srfAA*
srfAA-R	GCTTTGTCTCTTGCTTCTCCA	
ppsC-F	TGATGCTCTATTGGTTGGACTG	qRT-PCR for *ppsC*
ppsC-R	TAGCCCGATTCCGAAACA	
gyrB-F	GAAGCACGGACAATCACC	reference gene
gyrB-R	TCCAAAGCACTCTTACGG	
